# External ventricular drainage in modern neurosurgical practice: optimization, standardization, and emerging guidance technologies

**DOI:** 10.3389/fneur.2026.1807634

**Published:** 2026-05-05

**Authors:** Jack Hennen, Joseph Ifrach, Iris Charcos, Charles Morse, Manisha Koneru, Corey Mossop

**Affiliations:** 1 Department of Medicine, Rowan-Virtua School of Osteopathic Medicine, Stratford, NJ, United States; 2 Department of Neurosurgery, Cooper University Hospital, Camden, NJ, United States

**Keywords:** augmented reality, external ventricular drain, image guidance, navigation, standardization, ventriculostomy

## Abstract

External ventricular drain (EVD) placement is a standard neurosurgical procedure for cerebrospinal fluid diversion and intracranial pressure monitoring, yet conventional freehand techniques are associated with substantial variability in placement accuracy and complication risk. Anatomical distortion and ventricular compression commonly seen in the setting of pathology further limit the landmark-based reliability of freehand drain insertion, increasing the likelihood of multi-pass attempts and suboptimal catheter placement. Consequently, adjunct guidance technologies, including ultrasound, electromagnetic neuronavigation, and augmented reality (AR) platforms have been developed to improve procedural precision and consistency. This review examines current evidence comparing freehand and technology-assisted EVD placement, integrating an overview of the technical principles underlying contemporary guidance systems with a synthesis of comparative clinical outcomes across modalities. Reported clinical evidence suggests guided techniques can improve catheter placement accuracy and first-pass success, while workflow impact varies by modality and institutional experience. Electromagnetic navigation demonstrated consistent performance across settings, with AR platforms representing an emerging approach with potential advantages in portability and ease of integration. Continued efforts toward procedural standardization and prospective validation may further define the role of guided techniques in advancing patient safety and reducing variability in ventriculostomy practice.

## Introduction

External ventricular drain (EVD) placement is a commonly performed neurosurgical procedure used to provide therapeutic CSF diversion and diagnostic intracranial pressure (ICP) monitoring in patients with traumatic brain injury (TBI), hydrocephalus, intracerebral hemorrhage (ICH), and intraventricular hemorrhage (IVH) among other causes of increased intracranial pressure. Despite its ubiquity in neurocritical care, ventriculostomy can be technically challenging in the setting of distorted anatomy, and insertion technique may vary by institution and operator (1, 2).

The conventional approach is done freehand, utilizing surface anatomical landmarks, most commonly Kocher’s point, to guide the catheter tip into the ipsilateral foramen of Monro (2–4). Reported placement accuracy in freehand techniques is subject to substantial variability, and suboptimal catheter placement is not uncommon (4–6). Misplacement and multiple-pass attempts have been found to significantly increase risk for complications including catheter obstruction, post-operative hemorrhage, infection, and catheter dislodgement (5, 7, 8). The difficulties of accurate catheter placement are often exacerbated in emergent settings due to atypical anatomy and potential midline-shift, making the traditionally-used landmarks of freehand placement less reliable (9, 10).

Aligning with broader trends toward precision-guided neurosurgery, such as integration of image guidance in spinal instrumentation and neurointerventional procedures, the persistent variability and risks associated with ventriculostomy have driven the adoption of assistive technologies including ultrasound assistance, neuronavigation, electromagnetic stereotaxy, and, more recently, augmented reality (AR) platforms (11–13).

Beyond demonstrating enhanced accuracy, institutional experiences have shown image-guided systems to reduce complication rates. While workflow integration initially adds setup time for registration and planning, actual operative time remains comparable to traditional freehand methods, and streamlined protocols can minimize procedural delays (14–16). Adoption of these techniques across institutions remains inconsistent due to differences in resources, training requirements, workflow integration challenges, and perceived clinical benefit (17, 18).

The purpose of this review is to synthesize the current evidence regarding conventional freehand and technology-assisted EVD placement, to outline the mechanisms and performance of contemporary navigation platforms, and to discuss their impact on accuracy, safety, and workflow. Relevant literature was identified through PubMed, Scopus, and Embase searches supplemented by manual review of references from included articles. Search terms included “external ventricular drain,” “ventriculostomy,” “electromagnetic navigation,” and “augmented reality,” with both recent and foundational publications considered for inclusion. We also highlight efforts progressing toward procedural standardization and emerging guidance innovations that may shape the future of ventriculostomy across neurosurgical and neurocritical care settings.

## Overview of EVD technique and adjunct guidance technologies

### Conventional freehand approach

The conventional freehand approach remains the most widely practiced method for EVD placement globally and makes use of external anatomical landmarks to plot the ideal trajectory to the optimal placement site at the ipsilateral frontal horn of the lateral ventricle or the foramen of Monro. Kocher’s point, located 2–3 cm lateral from the midline at the mid-pupillary line and 1–2 cm anterior to the coronal suture, approximately 11 cm from the glabella, is the most commonly used entry site in modern practice (17). Notable alternatives include Frazier’s point (6 cm above and 4 cm lateral to the inion) and Keen’s point (2.5–3 cm posterior and 2.5–3 cm above the pinna), which provide parieto-occipital access when frontal approaches are contraindicated (19).

### Ultrasound-guided approach

Intraoperative ultrasound guidance provides an alternative to landmark based placement through direct real-time visualization of ventricular anatomy without requiring preoperative imaging or image registration. First described in 1981 for ventriculoperitoneal shunt placement in infants, ultrasound guidance has since been adapted for use in EVD placement across age ranges (20, 21). The technique employs specialized burr hole probes with integrated needle guide channels that display a virtual trajectory line on the ultrasound image, enabling operators to advance the catheter under continuous visualization while adjusting tip position away from the choroid plexus (21, 22). To permit simultaneous catheter placement and positioning of the transducer, operators may either expand the standard burr hole or drill an overlapping “spectacle” burr hole at the selected point of entry (4, 21, 22). However, limitations including equipment availability, operator learning curves, and technical challenges of simultaneous probe manipulation have stalled the more widespread adoption seen with other image guided techniques (22).

### The role of image-based stereotactic guidance

Image-guided systems align the preoperative imaging dataset with the patient’s physical anatomy through registration methods including surface-based scanning, fiducial markers, or electromagnetic tracking, the latter detecting sensor-equipped instruments within a generated field eliminating the need for a direct line of sight (23–26). Accuracy is influenced by factors such as image resolution, registration error, and intraoperative factors such as brain shift and patient movement, highlighting the importance of optimized imaging protocols and continuous referencing throughout the procedure (25–28).

**Figure 1:**
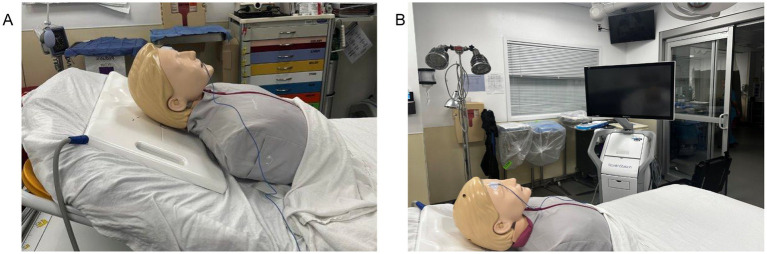
Stealth station set up. **(A)** Patient positioning with the flat electromagnetic field emitter placed beneath the head. **(B)** Bedside positioning of the StealthStation navigation workstation for electromagnetic-guided ventriculostomy.

**Figure 2:**
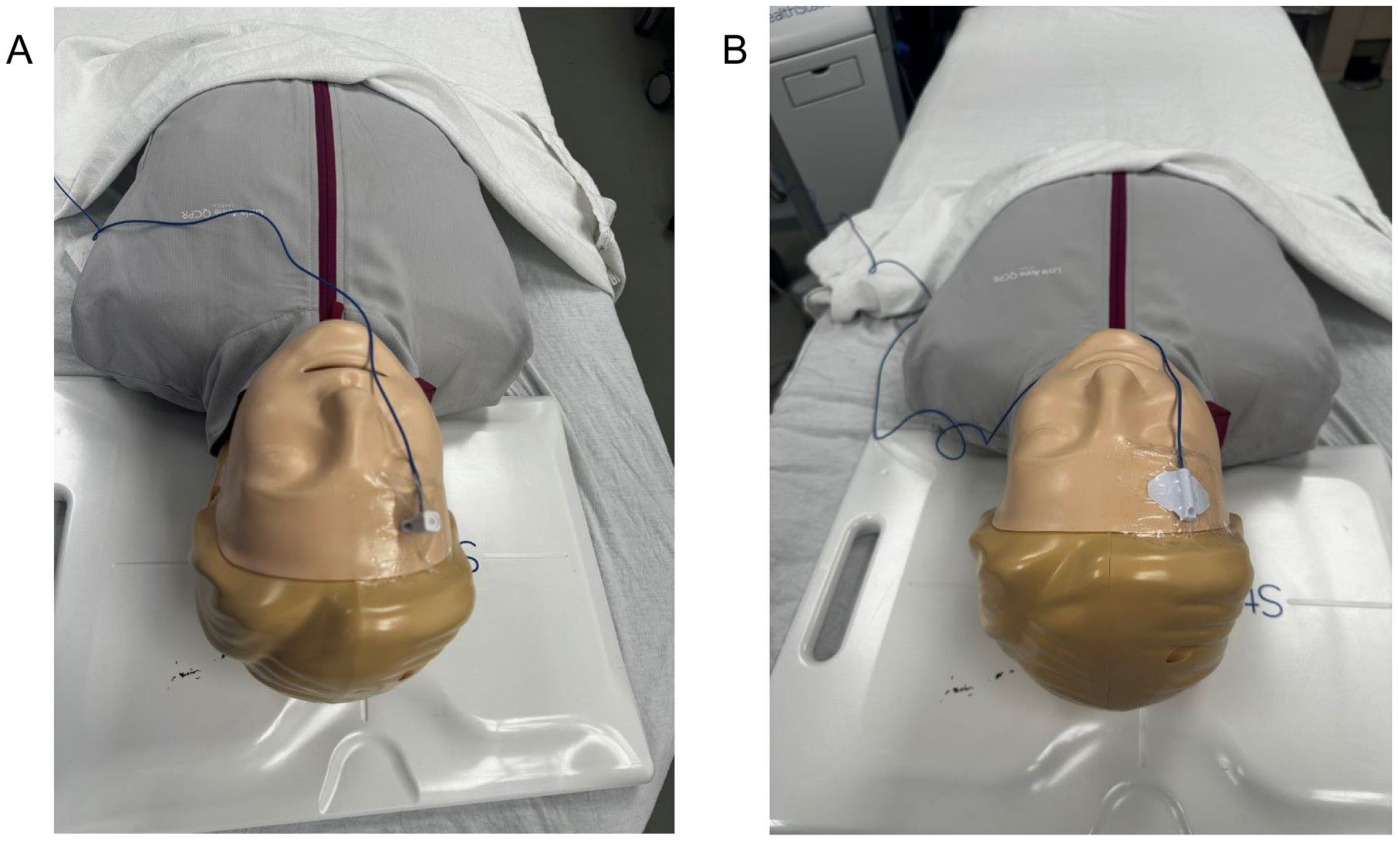
Navigation tracker placement. **(A)** Placement of a skull-mounted electromagnetic navigation tracker secured using an adhesive dressing. **(B)** Placement of a noninvasive electromagnetic navigation tracker affixed to the scalp.

Contemporary electromagnetic platforms such as the StealthStation AxiEM (Medtronic, Inc.) generate an electromagnetic field around the patient’s head that detects sensors embedded within the catheter stylet and a stationary reference tracker affixed to the patient (26, 29, 30). The navigation workstation continuously maps the three-dimensional position of these instruments onto thin-slice preoperative CT datasets acquired at admission, providing real-time trajectory visualization throughout the procedure. Using a portable cart positioned at the bedside, the electromagnetic field emitter is placed beneath the patient’s head and the non-invasive tracker is applied and registered to the imaging dataset (14, 16) (Figures 1, 2). Surgical planning identifies Kocher’s point as the entry site and the ipsilateral foramen of Monro as the target, with live stereotactic feedback guiding stylet and catheter advancement along the planned trajectory. Optical tracking systems, despite having demonstrated reliable accuracy in a number of neurosurgical subspecialties, are less ideally suited for bedside ventriculostomy placement due to requiring rigid head immobilization and maintenance of an unobstructed line of sight (31–33).

### Augmented reality guidance

Augmented reality guidance represents a paradigm shift in surgical navigation by superimposing computer-generated anatomical reconstructions and planned trajectories directly onto the surgeon’s field of view, integrating preoperative imaging data into the real-world surgical environment in real time (34–36). Unlike conventional navigation systems that require visual reference to external monitors, AR platforms project patient-specific holographic overlays via head-mounted displays, microscope heads-up displays, or tablet-based interfaces, enabling hands-free visualization while maintaining direct sight of the surgical field (36, 37).

**Figure 3:**
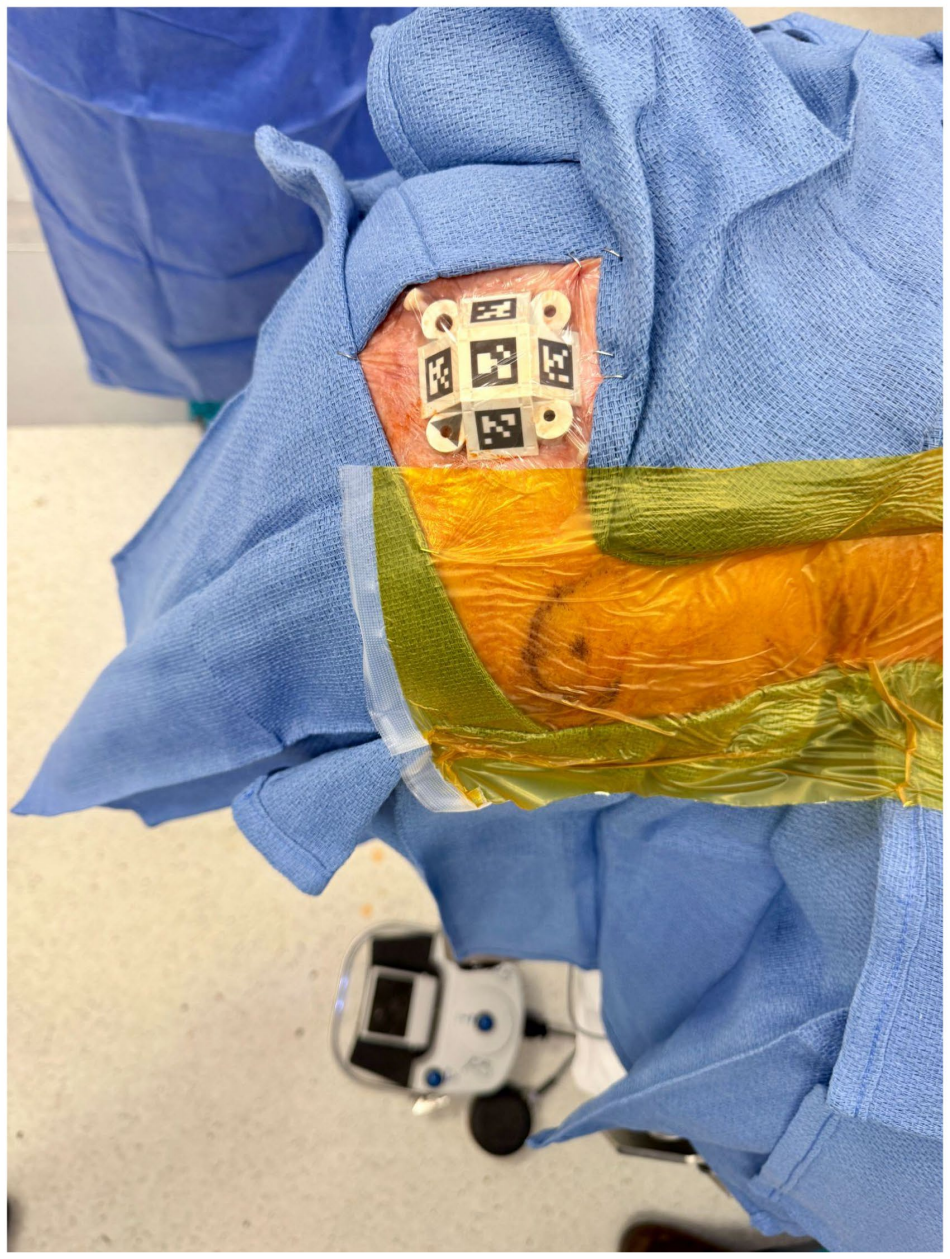
AR tracker placement. Placement of the augmented reality registration tracker on the patient’s head using an adhesive solution (e.g., Mastisol).

**Figure 4:**
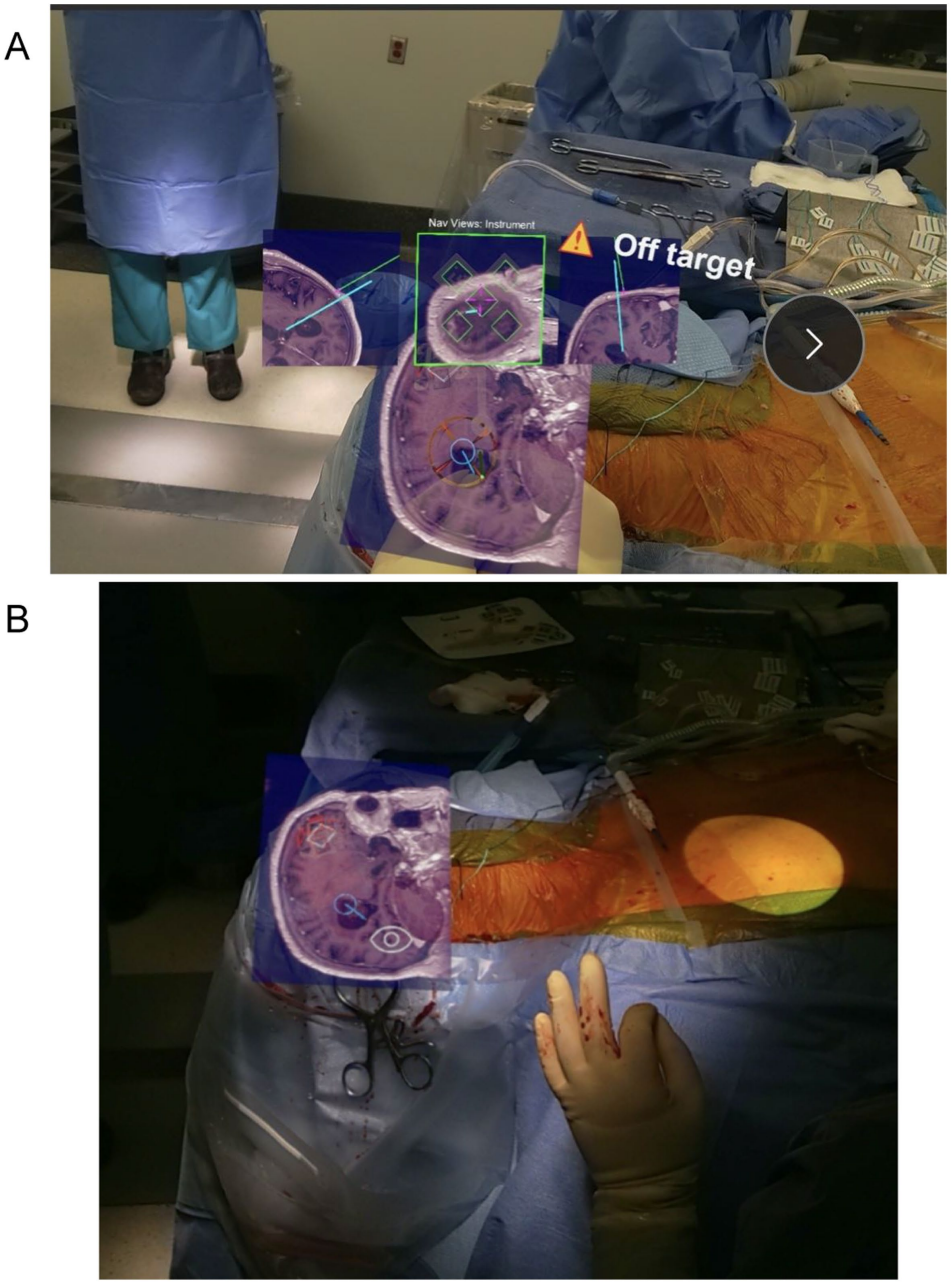
Perspective from augmented reality device. **(A)** Augmented reality headset view with overlaid axial imaging and planned catheter trajectory during ventriculostomy planning. **(B)** Augmented reality headset view showing real-time visualization of patient anatomy and catheter trajectory during ventriculostomy placement.

AR systems align virtual overlays with patient anatomy through registration methods including surface scanning, fiducial markers, or cranial landmark-based alignment, with markers affixed to the patient’s forehead providing stable reference frames for the continuous spatial registration (35–37) (Figure 3). The holographic overlay dynamically updates in real time to maintain spatial fidelity as the procedure progresses, accommodating minor patient movements or anatomical shifts without requiring re-registration (35) (Figure 4).

**Figure 5:**
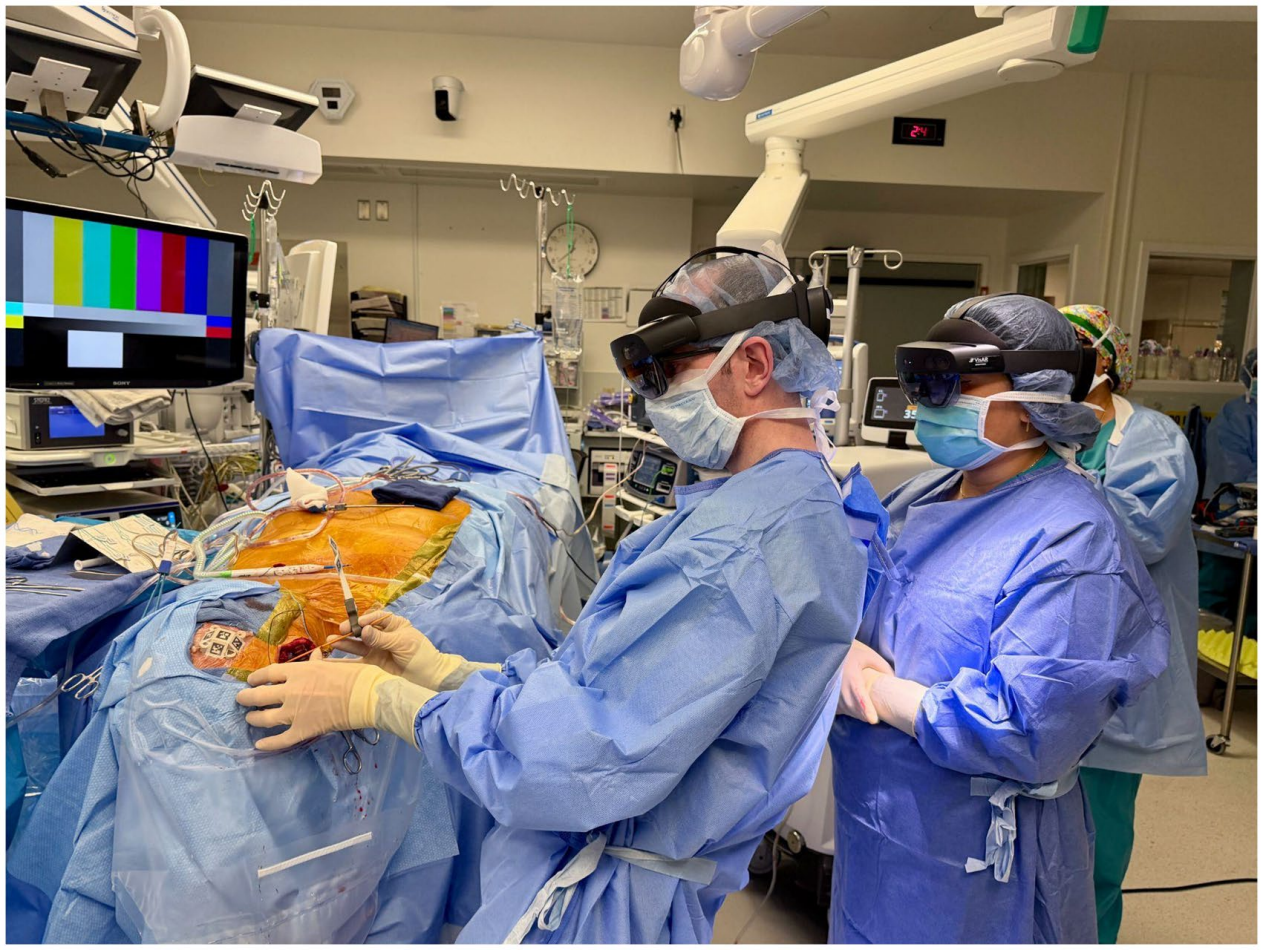
AR placement of ventriculostomy catheter. Augmented reality-assisted setup and catheter trajectory visualization during ventriculostomy placement.

Key technical advantages over optical tracking systems include elimination of line-of-sight requirements between cameras and tracked instruments, hands-free visualization with holograms superimposed directly on the surgical field, streamlined workflow in space-constrained bedside environments, and enhanced portability for critical care settings (34–36) (Figure 5).

Broader neurosurgical experience with AR has demonstrated improved tumor resection rates, enhanced preservation of neurologic function in eloquent cortex procedures, and utility as a surgical training tool, lending context for its expanding application to EVD placement (38–41).

In their current iteration, AR systems also have some notable limitations. Setup time is variable and system dependent, especially as compared to bedside electromagnetic navigation. There is a significant learning curve to using these systems as workflows are disparate and not particularly user friendly.

## Current clinical evidence for adjunct guidance in EVD placement

### Comparative accuracy: freehand vs image-guided techniques

Early bedside series analyzing freehand EVD placement accuracy found substantial variability in optimal catheter positioning, with Kakarla et al. reporting optimal placement (Grade 1) in 77% of cases, functional but suboptimal placement (Grade 2) in 10%, and non-functional or eloquent cortex placement (Grade 3) in 13% (7). Of note in their study encompassing 346 patients, rates of suboptimal placement were significantly higher in those with traumatic brain injury (*p* = 0.0001) and midline shift (*p* = 0.059) while optimal placement rates were highest in subarachnoid hemorrhage patients (*p* = 0.003), highlighting the technical challenges posed by anatomical distortion as it pertains to freehand EVD placement (7). Subsequent meta-analyses have corroborated these findings, with Nawabi et al. (5) pooling 6,313 EVDs placed via freehand technique across 39 studies and reporting 72% optimal placement (95% CI: 66–77%), 78% first-pass success (95% CI: 67–86%), and complication rates of 7% for hemorrhage and 5% for infection. Stuart et al. (6) analyzed 2,983 cases from 19 studies reporting Kakarla grading and found a mean ideal placement rate of 73% (SD ± 7%), noting substantial heterogeneity in outcome measures and reporting quality across institutions.

Ultrasound-guided EVD placement has shown improved accuracy in several comparative studies. Zhang et al. (21) compared 131 ultrasound-guided catheter placements with 59 freehand insertions in patients with severe IVH, and reported optimal placement in 88.55% of ultrasound-guided cases compared to 42.37% with freehand technique (*p* < 0.001). Operation and catheterized times were found to be increased among the ultrasound group, but complication rates did not differ from the control group (23). Fisher et al. (4) evaluated 294 EVD patients including 45 ultrasound guided procedures and found suboptimal placement rates of 15.6% with ultrasound versus 20.8% freehand, although this did not reach statistical significance (*p* = 0.168). The ability to provide real-time visualization has been reported as particularly useful when rapid bedside access is needed in patients with compressed or shifted ventricular anatomy (4, 21, 42).

A number of comparative trials have systematically evaluated the effectiveness of electromagnetic navigation to address the limitations of freehand placement. AlAzri et al. (43) compared 54 severe traumatic brain injury patients, with all prospective cases over 1 year receiving electromagnetic navigation (*n* = 19) versus retrospective freehand controls (*n* = 35), and reported optimal placement rates of 94.7% versus 57.1% (*p* = 0.009), with mean number of passes significantly reduced in the navigation group (1.16 ± 0.38 versus 1.63 ± 0.88, *p* = 0.018). Shtaya et al. (15) retrospectively analyzed 331 ventriculostomies (44 image-guided, 287 freehand) and demonstrated that image guidance improved optimal placement rates from 60.6 to 75% in unmatched cohorts (*p* = 0.067). After propensity score matching, the image-guided group achieved significantly higher optimal EVD placement than freehand (75% vs. 43.2%; OR 4.6), with greatest benefit in patients with smaller ventricles (Evans index < 0.36), while no significant advantage was seen in those with larger ventricles. Overall complications and revision rates were lower with image guidance, and no significant increase was seen in operative time (15). Nielsen et al. (17) retrospectively compared 86 electromagnetic-guided EVD placements with 163 freehand insertions and found higher rates of Kakarla Grade 1 positioning with electromagnetic guidance (93.0% vs. 84.0%, *p* = 0.044), along with greater first-pass success (90.7% vs. 86.5%). Notably, electromagnetic guidance was preferentially used in cases with greater degrees of anatomical complexity, such as in patients with significantly smaller ventricles, increased midline shift, and lower Evans indexes, yet still achieved higher rates of accuracy (17). In contrast, McLean et al. (9) analyzed 632 EVD insertions across 21 tertiary centers in the UK and Ireland and found that image guidance (utilized in only 19.6% of cases) did not significantly improve catheter tip position or reduce drain blockage rates, even when stratified by ventricular size. Early electromagnetic navigation studies raised concerns regarding procedural efficiency. Mahan et al. prospectively enrolled 35 consecutive ICU patients and reported that electromagnetic navigation achieved 94.3% optimal placement with only one tract hemorrhage, though total procedural time (including setup and registration) increased by 36 min (16).

At our institution, outlined in Charcos et al. (14), electromagnetic stereotactic navigation has been incorporated into bedside EVD placement within trauma and neurocritical care units, with dedicated Medtronic StealthStations available for rapid deployment. Image registration uses existing diagnostic CT datasets and a noninvasive reference tracker, which is secured under sterile conditions. With this optimized workflow, navigated placement produces high accuracy without prolonging procedural time. In fact, median total procedural durations were similar between guided and freehand techniques (approximately 55 vs. 60 min, *p* = 0.71) despite more difficult anatomic variance in navigated cases. In retrospective comparison, electromagnetic guidance achieved 100% optimal placement and single-pass success, compared with 88.2% optimal placement and 73.5% single-pass success in freehand insertions. Importantly, attributable risk reduction analyses indicate that for every nine navigated EVDs performed, one additional suboptimal placement is prevented, and for every four navigated EVDs, one additional multi-pass attempt is avoided. This risk reduction indicates clinically interpretable benefits of navigation in both quality and efficiency. These findings suggest that image-guided EVDs can significantly improve accuracy, particularly when ventricular anatomy is distorted, without imposing meaningful delays on care.

Augmented Reality platforms represent the most recent technological evolution in EVD guidance, while optical tracking systems have seen limited clinical adoption for EVD placement in published literature, likely due to line-of-sight constraints and workflow integration challenges in emergent bedside scenarios. Li et al. reported the first clinical trial of AR-guided EVD placement in 15 patients and demonstrated significant reductions in mean catheter passes (1.07 ± 0.26 versus 2.33 ± 0.98, *p* < 0.01) and trajectory deviation (4.34 ± 1.63 mm versus 11.26 ± 4.83 mm, *p* < 0.01) compared to freehand placement in 15 retrospectively analyzed cases, although on average 40.20 ± 10.74 min were added on before the procedure to account for planning and registration (34). Van Gestel et al. (44) subsequently demonstrated workflow maturation in a prospective pilot study of 11 AR-guided placements compared to 11 freehand controls, achieving 100% first-attempt functional placement versus 64% for freehand (*p* = 0.045) and 73% optimal placement versus 27% (*p* = 0.043). No AR-guided placements required revision, whereas the freehand group had a 36% reintervention rate (*p* = 0.045), and procedure-related complications occurred in 18% of AR cases versus 45% in freehand controls (18). Registration and trajectory planning required approximately 2 min and procedure time in the AR group was reportedly around 15 min in total (44).

The collective evidence shows that electromagnetic and augmented reality guidance systems improve accuracy compared to freehand placement, particularly in patients with pathological distortions of anatomy (14, 15, 43, 44). Electromagnetic guidance systems have been more widely integrated at present, and initial workflow integration difficulties have been largely overcome, while AR platforms show promising early results but will require refinement of registration workflows in addition to larger-scale validation to gain an established role in routine clinical practice (14, 16, 30, 44). Beyond EVD-specific applications, broader experience with AR guidance in neurosurgery provides important context for understanding the technology’s potential trajectory and challenges to integration. Similar real-time three-dimensional guidance approaches have been investigated for catheter placement in ventriculoperitoneal shunt surgery, suggesting that technologies that enable real-time trajectory visualization may broadly improve ventricular access procedure accuracy (45).

### Areas of standardization in EVD protocols and research

Assistive technologies improve upon freehand ventriculostomy placement. As image-guided and augmented reality techniques mature, parallel efforts toward protocol standardization will be essential to ensure that technical advances translate into reproducible clinical benefit across institutions (1, 3).

Despite the EVD’s central role in neurocritical care, protocols for EVD placement, management, and outcome reporting vary across institutions, limiting comparability and consensus development. Infection prevention and catheter care remain critical targets for standardization. Evidence supports the use of tunneled catheters, antibiotic-impregnated EVDs, closed-system handling, and structured care bundles to reduce ventriculitis risk (1). Multiple-pass insertion attempts were found to be correlated with increased risk infection and hemorrhage, underscoring the importance of first-pass accuracy as both a procedural quality metric and an infection prevention strategy (5, 8). Duration of catheter indwell demonstrates a positive association with infection risk, with evidence suggesting increased rates beyond the first week, although the relationship may not be strictly linear (1, 46). Institutional adherence to EVD placement and maintenance protocols has been shown to achieve low infection rates independent of catheter duration, while catheter replacement through the same access tract appears to confer persistent heightened risk of EVD related infection (46).

Monitoring, weaning, and outcome reporting likewise remain heterogeneous across centers. Drainage protocols differ regarding continuous versus intermittent CSF diversion, target pressure settings, weaning strategies, and timing of catheter removal (47–49). Comparative studies and systematic reviews suggest that different weaning strategies involve trade-offs between clinical stability, length of hospital stay, and risk of treatment failure, but heterogeneity in protocols and outcome definitions has limited the ability to establish definitive best practices (47–49).

Emerging technologies, including automated cerebrospinal fluid drainage systems with integrated pressure and flow monitoring, offer additional opportunities to reduce management variability and support more objective, reproducible EVD care (50). However, heterogeneity in outcome definitions and analytic approaches across EVD placement studies continues to limit direct comparability (6). Greater standardization of placement-related endpoints and reporting frameworks will be essential to accurately assess the clinical impact of image-guided and augmented reality techniques.

## Challenges, barriers, and future directions

### Barriers to adoption

Despite growing interest in image-guided and augmented reality assisted EVD placement, several barriers continue to limit widespread adoption. Cost considerations remain a major factor, including initial capital investment, ongoing maintenance, and institutional resource allocation. Workflow integration and the need for efficient image registration also present challenges, as navigation systems must be deployable rapidly in emergent settings without introducing procedural delays or excessive setup complexity (16, 34). Successful implementation is also dependent on operator training and familiarity, particularly for technologies that deviate from traditional landmark-based approaches. Lastly, the increasing incorporation of digital overlays, image transfer, and data integration raises regulatory, cybersecurity, and patient privacy considerations that must be addressed as these systems scale (51).

### Evidence gaps and research needs

Current evidence supports the premise that image-guided EVD placement improves accuracy compared with freehand techniques, with electromagnetic navigation serving as a well-validated reference standard and augmented reality demonstrating technical feasibility, important gaps in the literature remain (5, 14, 15, 17, 43, 44). Comparative effectiveness between neuronavigation systems have not yet been fully elucidated, and long-term outcomes, cost-effectiveness, and optimal patient-selection criteria remain incompletely defined (30). In addition, training for image-guided techniques remains largely simulator-based without standardized curricula or competency assessments (18, 41). Addressing these gaps will require well designed, multicenter prospective trials with harmonized outcome measures and placement-focused endpoints.

Our group has planned a trial to assess the efficacy and accuracy of the different imaging modality adjuncts available for EVD placement. The PEARL trial (Placing External Ventricular Drains Using Assistive Augmented Reality or Image-Based Localization; NCT07042048) is a retrospective, single-center propensity matched cohort analysis to evaluate AR-assisted EVD placement compared to electromagnetic image-guided stereotactic navigation. The study will enroll 50 patients (25 per arm) that require EVD placement for spontaneous intracerebral hemorrhage with associated intraventricular hemorrhage or severe traumatic brain injury. The primary endpoint assesses optimal catheter tip placement as demonstrated on post-procedure CT imaging and a determined Kakarla Grading Score by masked assessors. Secondarily, we will evaluate the number of passes, procedure time, accuracy and related complications such as hemorrhage, occlusion, dislodgement, and infection. The overall objective of the trial will be to determine whether AR-assisted EVD placement is non-inferior to electromagnetic stereotactic assisted placement in accuracy and safety, while potentially offering advantages in portability, efficiency and ease of use.

At this time, bedside freehand EVD placement remains the standard while stereotactic navigation represents the benchmark for precision and reproducibility. The development of AR provides a natural extension of the same paradigm with the goal of achieving equivalent or even superior spatial acuity while reducing equipment burden and streamlining workflow. These technologies together progress the field toward a more standardized guided and trainable procedure, enabling consistent procedural quality across operators and institutions. Looking ahead, anticipated developments include AI-driven trajectory planning, hybrid AR-stereotactic systems and remote visualization platforms for simulation and real-time procedural support. The PEARL trial will be the first expected trial evaluating head-to-head validation of AR accuracy, safety and workflow efficiency for ventriculostomy.

### Potential future innovations

Future innovations in EVD placement and management are likely to build on advances in computational guidance and device integration. Artificial intelligence-assisted trajectory planning algorithms have demonstrated technical feasibility in autonomously identifying optimal entry points and target coordinates on clinical imaging, including in cases with intracranial hemorrhage (52). Cloud-enabled navigation platforms could facilitate rapid image transfer, remote support, and procedural standardization across institutions. In parallel, the development of closed-loop EVD catheters incorporating pressure and flow sensing with integrated data dashboards may enable more objective monitoring and tighter linkage between placement accuracy and downstream management decisions.

## Conclusion

External ventricular drain placement remains an essential but variably performed procedure in neurosurgical and neurocritical care practice. Advances in image-guided techniques, such as electromagnetic navigation and augmented reality platforms, offer meaningful opportunities to enhance placement precision, reduce variability, and promote workflow standardization. Continued efforts in protocol harmonization, outcome standardization, and prospective validation will be necessary to ensure that these advances translate to sustained improvements in patient care.
